# Muco-Obstructive Lung Disease: A Systematic Review

**DOI:** 10.7759/cureus.46866

**Published:** 2023-10-11

**Authors:** Garima Singh, Sourya Acharya, Samarth Shukla, Dhriti Jain

**Affiliations:** 1 Medicine, Jawaharlal Nehru Medical College, Datta Meghe Institute of Higher Education and Research, Wardha, IND; 2 Pathology, Jawaharlal Nehru Medical College, Datta Meghe Institute of Higher Education and Research, Wardha, IND

**Keywords:** non-cystic fibrosis bronchiectasis, muco ciliary clearance, primary ciliary dyskinesia, cystic fibrosis, copd, infection, obstructive lung disease, respiratory tract, mucus

## Abstract

Muco-obstructive lung disease is a new classification under the diseases of respiratory tract. A lot of discussion is still going on regarding this new group of diseases. It is characterised by obstruction of the respiratory tract with a thick mucin layer. Usually in normal individuals, the mucus is swept out of the respiratory system while coughing in the form of sputum or phlegm, but if the consistency of the mucus is thick, or the amount is heavy or there is a certain defect in the ciliary function of the respiratory tract, the mucus is not cleared and it gets accumulated in the lungs alveoli, therefore blocking it. The mucus trapped in the distal airways cannot be cleared by coughing therefore forming a layer in the alveoli and bronchioles. Long-standing condition causes inflammation and infection. This new group of diseases specifically includes chronic obstructive pulmonary disease (COPD), cystic fibrosis (CF), primary ciliary dyskinesia (PCD) and non-cystic fibrosis bronchiectasis (NCFB). Asthma, although an obstructive disease of the lung, is not particularly included under muco-obstructive lung disease. The major symptoms with which these diseases present are sputum production, chronic cough and acute exacerbations of the condition. The mucus adheres to the lung parenchyma causing airway obstruction and hyperinflation. In this article, we will see how muco-obstructive lung diseases affect the normal physiology of the respiratory system and how is it different from other obstructive and restrictive lung diseases. We will individually look into all the four conditions that come under the category of muco-obstructive lung diseases.

## Introduction and background

The group of illnesses known as muco-obstructive lung disorders affects the airways and damages the lungs structurally over time; cystic fibrosis (CF), chronic obstructive pulmonary disease (COPD), primary ciliary dyskinesia, non-cystic fibrosis bronchiectasis are a few examples of muco-obstructive lung disease that affect the airways and progressively damage the structure of the lungs [1-4].

In the case of a healthy person, the mucus layer is well-hydrated and is rapidly transported to the trachea from the distal airways. When a person is affected with muco-obstructive disease, epithelial defects in ion fluid transport, secretion of mucus, or both of them together lead to hyper concentration of the mucus, forming dehydrated mucus, failure of transport of mucus to the trachea, and adhesion to the airway surfaces. If mucus accumulates in the trachea, it can be cleared by cough as sputum or phlegm. But mucus accumulated in the small airways cannot be removed, resulting in airway obstruction and making it vulnerable to infection and inflammation [5]. Even though numerous researches have concentrated on the pathophysiology of muco-obstructive illnesses, there is a vast but unmet demand for efficient therapeutic options because the progression and genesis of many diseases are complex and not properly understood to date.

Disease heterogeneity

It implies that a few regions in the lungs are unaffected and other areas of the ipsilateral side are severely diseased. This is the key characteristic of muco-obstructive lung disorders. An early manifestation of disease that occurs in the tiny airways, i.e. the bronchioles, as shown by micro-computed tomography, pathological examination and pulmonary-function investigations, is a characteristic that unifies muco-obstructive disorders. It's unlikely that a slowdown in the mucus clearance alone can cause disease [6].

For muco-obstructive illness to manifest, there must be deposition of the mucus layer, resulting in the development of mucinous plugs and plaques within airway lumens. The whole spectrum of muco-obstructive disorders, such as airway blockage, inflammation, and intermittent infection, have been shown in animal models of these diseases, not just impairments in mucociliary clearance alone, according to these studies [7]. The development of these plugs of mucus or the plaques in the airways results from increased mucin secretion, which is frequently triggered by infections and inadequate epithelial hydration of newly released mucins. A typical aspect of muco-obstructive lung disorders is bacterial infection [2-4, 8]. Clinically, a pulmonary exacerbation is manifested as increased respiratory symptoms such as cough and sputum production, often accompanied by systemic symptoms such as anorexia and malaise [9]. The severity and frequency of acute exacerbations can most likely significantly impact the overall development rate of the disease's severity in all muco-obstructive lung diseases. It's vital to remember that an exacerbation is a condition that develops on top of a variety of muco-obstructive lung diseases. Researches suggest that certain exacerbations are brought on by a worsening of the disease in locations where it already exists, maybe due to genetic drift or phenotypic responses to the local environment [10-11].

Methodology

**Figure 1:**
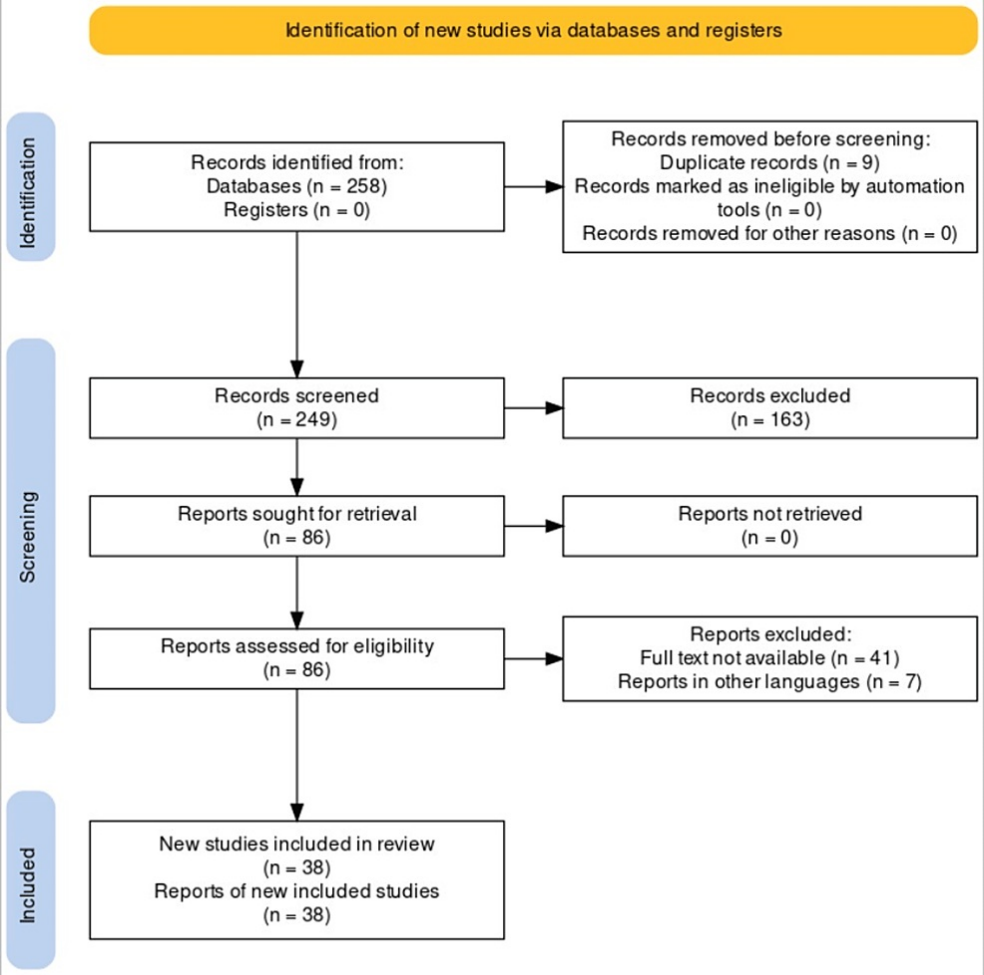
PRISMA flow diagram of muco-obstructive lung diseases Adopted from the Preferred Reporting Items for Systematic Reviews and Meta-Analyses (PRISMA).

A systematic search was undertaken through PubMed in June 2023; keywords used for this review article were “lung diseases”, “muco obstructive lung diseases”, “obstructive lung diseases”, “mucociliary clearance”, ((Lung diseases) AND (muco obstructive lung diseases) AND (obstructive lung diseases) AND (muco ciliary clearance)). Some contents of this review article were taken from the key references of their respective bibliographies and citations. This review article was updated in August 2023. The inclusion criteria consisted of all articles that were published in the last five years which discussed muco-obstructive lung disease for which PubMed or the publisher provided open access. The articles that were excluded were not retrievable and discussed either because of written in some other language or the full text was not available. A total of 258 articles were found, but only 38 of them were chosen to be included because it was determined that they were pertinent. These were selected following the Preferred Reporting Items for Systematic Reviews and Meta-Analysis (PRISMA) guidelines. A comprehensive outline of the selection method is given in Figure 1.

## Review

Cystic fibrosis

In terms of the two-gel hypothesis defining a disease that is dependent on mucus concentration, cystic fibrosis is the condition that has been investigated the most. The fundamental impairment of the transport of airway epithelial ions is seen in the mucus concentration in the lungs of cystic fibrosis patients [4]. Insufficient bicarbonate and chloride ions secretion, which is regulated by the CFTR (cystic fibrosis transmembrane conductance regulator) and by an intact route of sodium absorption, the epithelial lining of the airway is susceptible to the hyperabsorption of fluid in this condition. The first adult cases of cystic fibrosis with hyper-concentrated mucus were identified. According to measurements, more significant overall mucin concentrations and a larger solids percentage were found in the sputum. Adults with cystic fibrosis' upper airways were imaged using optical coherence tomography, and the results showed indications of hyperconcentration of the mucus, the slowed down mucociliary clearance, and, as the two-gel hypothesis anticipated, a decrease in the periciliary layer height [12]. Patients with cystic fibrosis have shown a higher total concentration of mucus than that in a healthy person, as seen on sputum measurement. As the mucin concentrations increase, changes in the viscoelastic properties of the layer emerge, slowing mucus clearance and resulting in stasis of mucus [13].

Chronic obstructive pulmonary disease (COPD)

Hyperconcentration of mucus has been noted in the samples taken from the lower airways in patients suffering from COPD, and high concentrations of mucus are also associated with decreased rates of mucociliary clearance seen in vivo [14]. Persistent airflow obstruction is the most significant and common manifestation of COPD, which is most frequently seen as a response to some inhaled environmental agents [1,15]. Data from a large cohort study of individuals with COPD says that smoking cigarettes is a significant trigger of COPD and is associated with increased mucus concentration [16]. According to correlations between high concentrations of mucus and worsening obstruction of the airflow and rates of exacerbations, the mucin hyperconcentration in the sputum was linked to disease severity in this cohort [16]. Increased mucin concentrations were also seen in patients with practically normal lung function having early COPD who were demonstrated to be at risk of rapid progression of the disease.

The mucus hyper concentration caused by the airway epithelial abnormalities in COPD is complex. Limitation of hydration responses of paracrine adenosine-CFTR (cystic fibrosis transmembrane conductance regulator) that precede mucus production, hypersecretion of mucus is due to rapid metabolism of co-released adenosine diphosphate (ADP), adenosine monophosphate (AMP) and impaired CFTR combined adenosine. This may be particularly significant in COPD [17]. Perhaps, the mildest example of a muco-obstructive disease is the mucin hyper concentration that characterises COPD patients. Due to the reduced prevalence of bronchiectasis and pseudomonas infection, the least severe form of muco-obstructive pulmonary illness is most likely to be seen in patients with COPD [18,19].

Primary ciliary dyskinesia

The pathophysiology of primary ciliary dyskinesia has traditionally been viewed as being exclusively a motor problem brought on by abnormalities in the cilial shaft protein, cilial beat frequency, and impaired mucociliary clearance [3,20]. In primary ciliary dyskinesia patient's sputum is also abnormally hyper-concentrated, suggesting that there is also some other factor contributing to the aetiology of this disease [21]. The studies that revealed the mechano-transduction pathways connecting mucin concentration with cilial strain-induced release of ATP (adenosine triphosphate) also demonstrated that patients with primary ciliary dyskinesia had defects in this signalling pathway [22]. Due to this ciliary sensing error, less ATP is released into the airway surfaces, decreasing fluid secretion and forming a mucinous plaque that is hyper-concentrated or a mucinous clog that contributes to airflow restriction. This causes retention of mucus in the airway which promotes bacterial infection, further leading to exacerbations. Therefore deteriorating the quality of life, lung function and structure in patients with primary ciliary dyskinesia.

Non-cystic fibrosis bronchiectasis

**Figure 2:**
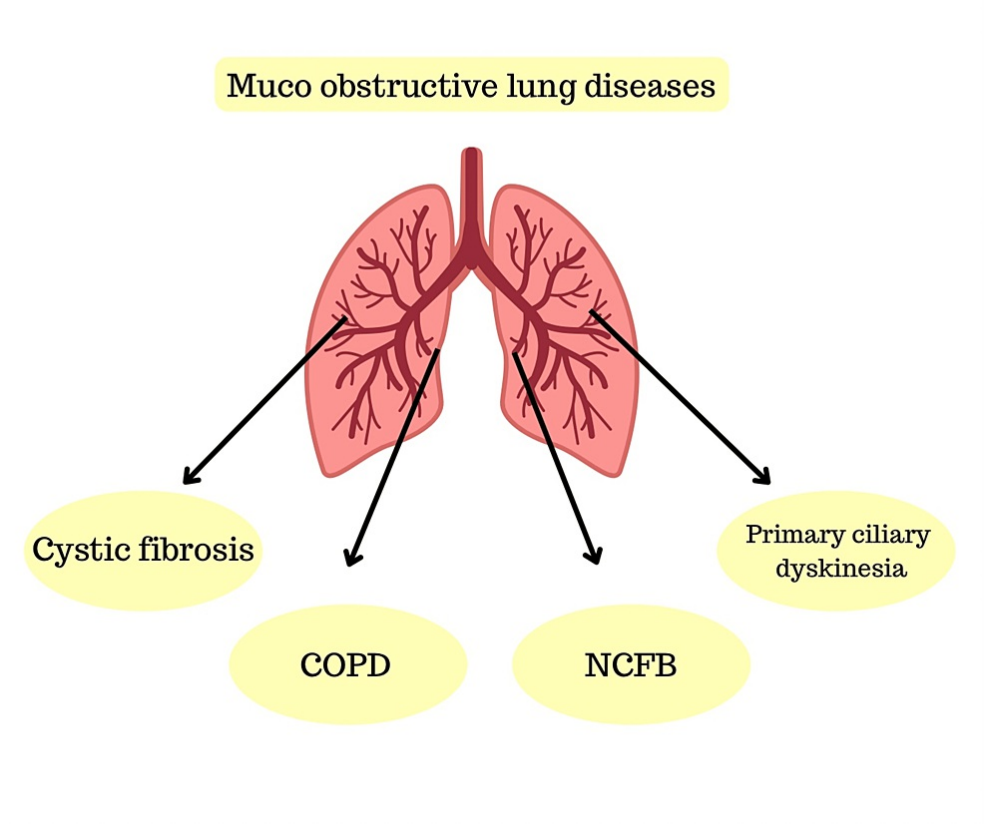
Diseases under muco-obstructive lung disease COPD: Chronic obstructive pulmonary disease; NCFB: Non-cystic fibrosis bronchiectasis Image credits: Garima Singh

Non-cystic fibrosis bronchiectasis can occur due to numerous factors which include primary immunodeficiencies, cilia abnormality, pneumonia and autoimmune diseases. Non-cystic fibrosis bronchiectasis is bronchitis without a single genetic aetiology [23]. Severe forms of small airway illness, which include mucus plugging, bronchiolectasis and inflammation, have been emphasised by classic pathological studies. Non-cystic fibrosis bronchiectasis has the usual muco-obstructive disease characteristic, i.e., impairment in airflow of the small airways, which is consistent with the above-mentioned pathological findings [24]. Non-cystic fibrosis bronchiectasis is commonly diagnosed on CT, which shows dilated bronchi on examination [25]. Recent observations showing non-cystic fibrosis bronchiectasis patients' hyperconcentration of mucus lead one to believe that underlying pathogenesis can be an error in the epithelium in controlling the hydration of the mucus. Approximately 15-20% of individuals with non-cystic fibrosis bronchiectasis have Pseudomonas aeruginosa positive microbiomes, which are typical of muco-obstructive illnesses and include staphylococcus, H. influenzae, and polymicrobial oral anaerobic bacteria [26]. Non-cystic fibrosis bronchiectasis resembles early cystic fibrosis in this situation. It is suggested that immune-deficiency alleles may play a role in non-cystic fibrosis bronchitis as the incidence of non-tuberculous mycobacterial infection in a patient with non-cystic fibrosis bronchiectasis can be up to 60% higher than that in patients with some other muco-obstructive disease [23,27]. Diseases under muco-obstructive lung disease are depicted in Figure 2.

Diagnosis

Each muco-obstructive lung illness has diagnostic criteria particular to that disease, such as genetic testing and sweat chloride anions for cystic fibrosis [28]; spirometry and history of any exposure in COPD [1]; for primary ciliary dyskinesia, measuring nasal nitric oxide, analysis of cilia waveform and genetic testing [3,20]; for non-cystic fibrosis bronchiectasis, CT scan can be done [23]. There are also resources available to provide a general diagnosis of muco-obstructive illness. General respiratory surveys can provide patient-related results relating to the occurrence of cough, sputum output, and exacerbation frequency. Sputum immune cells, cytokines, and chemokines can be measured to determine the inflammatory component of airway mucosal blockage. Additionally investigated as diagnostic tools are total mucin contents and mucus concentrations (percent solids) [16]. For instance, receiver-operating-characteristic curve analyses reveal that the concentration of mucus is a suitable biomarker for COPD since it is significantly correlated with chronic bronchitis symptoms as identified by patient-related outcome data [16]. The mucus hyper concentration caused by the airway epithelial abnormalities in COPD is complex. The limitation of hydration responses of paracrine adenosine-CFTR that precede mucus production, hypersecretion of mucus is due to the rapid metabolism of co-released adenosine diphosphate (ADP), adenosine monophosphate (AMP) and impaired CFTR combined adenosine. This may be particularly significant in COPD [17]. Perhaps, the mildest example of a muco-obstructive disease is the mucin hyper concentration that characterises COPD patients. Due to the reduced prevalence of bronchiectasis and pseudomonas infection, the least severe form of muco-obstructive pulmonary illness is most likely to be seen in patients with COPD [18,19].

Treatment

Disease-specific treatments are available for patients with cystic fibrosis. Ivacaftor (VX-770) is a potentiator of residual CFTR function in patients having gating of splicing mutations of the CFTR gene [29]. In vitro, hydration of airway surface and in vivo peripheral and central mucociliary clearance have an impressive association with clinically relevant outcomes that show ivacaftor played an essential role in developing therapies for muco obstruction. Cystic fibrosis patients who are homozygous for phenylalanine 508 deletion (F508del) in the CFTR gene can go with ivacaftor-lumacaftor and tezacaftor-ivacaftor as their treatment options. However, they have a less significant therapeutic benefit. Although they haven't received approval yet, corrector and potentiator three-drug combinations show promising results in patients who have at least one F508del mutation (which accounts for 90% of patients).

Rehydration

Rehydrating pathologic mucus in order to lower its concentration is arguably the most straightforward method of treating muco-obstructive lung illnesses. Inhaling osmotically active aerosols, such as mannitol or hypertonic saline, is the currently available method to accomplish this goal [30,31]. Development of epithelial Sodium Chloride (ENaC) targeted therapeutics is going on. ENaC inhibitors are in development as cystic fibrosis therapeutics to restore airway surface liquid hydration [32].

Hypertonic saline is the most frequently tried inhalational osmotic medication for muco-obstructive lung disorders. The best results of hypertonic saline were seen in cases of cystic fibrosis, which results in persistent increases in mucociliary clearance, higher FEV1 (forced expiratory volume) and decreased exacerbation rates [30,33]. The use of hypertonic saline inhalationally has been seen to be safe in inducing sputum in COPD patients but has not yet been tested therapeutically [34].

Mucolytics

Recent observations suggest that targeting the abnormal mesh and gel properties of the mucus, along with a reduction in concentration, has proven therapeutic. Firstly, the adhesive and cohesive properties of mucus are mainly governed by the viscosity of the mucus. Reduction in the thickness can be seen with mucin S-S bond reducing or surfactant agents. These agents help reduce the adhesive and cohesive properties of mucus irrespective of their concentration [35]. Secondly, mucus plaques obtained during bronchoalveolar lavage from patients with cystic fibrosis were said to be “permanent” gels because of their inability to dissolve even in excessive solvents [36]. Medications are being created that aim to lessen inter-mucin S-S bonds, such as surfactants or glycopolymers, to increase mucus swelling and cough clearance. The process of mucin reduction necessary for acetylcysteine to show improvement when inhaled has yet to be demonstrated [37].

Obstructive vs. restrictive lung disease

Obstructive Lung Problems

People with obstructive lung disease have shortness of breath because it's hard for them to exhale all the air from their lungs [38]. The characteristics of obstructive lung disease are slowed down, shallow, sluggish exhalations due to obstruction in the airways. Airway obstruction happens when the inflammation and swelling result in constriction of the airways or clogging them, making it difficult to remove the air from the lungs, resulting in large and excess amounts of air remaining in the lungs i.e., increased residual volume. Hyperinflation of the lungs and the entrapped air within worsen respiratory symptoms. These can present with symptoms like wheezing and excessive mucus production, or the lungs might also feel chronically full or partly complete.

Restrictive Lung Problems

People with restrictive lung disease can't fully fill their lungs with air. Their lungs are restricted from fully expanding [38]. Restrictive lung disorders are different from obstructive as the characteristics of restrictive disorders are problems in inhalational physiology. The hallmark of restrictive disorders is that the total capacity of the lung is reduced. The causes can be extrinsic, intrinsic or neurological in origin. Disorders that are restricted in the lungs (commonly referred to as "stiffening") are known as intrinsic restrictive disorders. Restrictive lung disorders can present as difficulty in inhaling enough air, which might also cause panic.

Obstructive and restrictive lung disorders have quite diverse treatment options, albeit these alternatives also rely on the specific root cause. The symptoms of obstructive lung illnesses, such as COPD and asthma, can be lessened by bronchodilators, which widen the airways. Steroids used orally or via inhalation are widely used to decrease inflammation. There are fewer alternatives for treating illnesses of the restricted lungs: Treatment of the underlying cause, such as a pleural effusion or ascites, may lead to improvement in extrinsic restrictive lung disease. The management of the condition may also be helpful in patients with intrinsic restrictive lung disease, such as pneumonia. Idiopathic fibrosis was difficult to treat until recently, but now there are medications that help lessen its severity. Supportive therapy, which includes extra oxygen, noninvasive ventilation (like CPAP or BiPAP), or mechanical ventilation, can be beneficial for both types of lung disorders. Medicines that relax these smooth muscles and improve airflow are called bronchodilators, and are inhaled [38]. People with COPD or those who have undergone surgery for lung cancer may benefit from pulmonary rehabilitation. Lung transplantation is another possibility when the condition is severe.

## Conclusions

Mucus hyperconcentration is a characteristic of muco-obstructive disorders. The four muco-obstructive illnesses have different epithelial defects that result in mucus hyper-concentration. Still, they all ultimately lead to the production of mucus plaques and plugs dependent on mucus concentration. Plaques of mucus adhered to the epithelial wall may trigger a muco-inflammatory loop with positive feedback, making these plaques permanent and eventually harming the airway walls. It makes sense to use treatments that aim to rehydrate and restore the viscosity or elasticity of mucus. The difficulty lies in getting these treatments to these small airways, i.e., bronchioles, which might already be occluded entirely due to the mucus, and the efficiency of deposition of aerosols might be subpar. However, research involving cystic fibrosis patients with mucus production reversed by potentiators gave rise to the idea that these diseases may be quite curable. Obstructive vs. restrictive lung disease prognosis is more influenced by the particular condition than by the kind of lung disease. Reversible obstructive lung illnesses frequently have a better prognosis than irreversible ones.
